# Future research of supply chain resilience: Network perspectives and incorporation of more stakeholders

**DOI:** 10.1016/j.fmre.2023.07.012

**Published:** 2023-11-15

**Authors:** L. Jeff Hong, Jinzhi Li, Xiaole Wu, Shuyue Yi

**Affiliations:** a School of Management, Fudan University, Shanghai 200433, China; b School of Data Science, Fudan University, Shanghai 200433, China

**Keywords:** Supply chain resilience, Supply chain networks, Bottlenecks, Global supply chains, Incentives design

## Abstract

In recent years, global supply chains face great challenges driven by events such as trade wars, the Covid-19 pandemic, and the Russia-Ukraine War. Since the disruptions caused by such shocks are more systematic and the duration is harder to predict compared to natural disasters or other operational risks, we highlight two features for future supply chain research: network perspectives and incorporation of more stakeholders such as governments that can influence overarching performances of industrial and supply chains. From the network perspective, we review the literature on vulnerable nodes & links identification, risk propagation modeling, network resilience measures, and disruption mitigation strategies. We suggest promising research directions such as deriving non-probabilistic resilience measures that can incorporate dynamics, extending existing methods for bottleneck detection to the network setting, balancing efficiency and resilience in a more sophisticated way, etc. In terms of incorporating more stakeholders’ roles in supply chain network decisions, we review the relevant literature and discuss research opportunities on governments’ roles in global supply chains (i.e., an external circulation perspective) and in incentivizing firms’ R&D and stimulating domestic demand (i.e., an internal circulation perspective). We suggest topics including global supply chain resilience evaluation using trade networks, technology networks, and firm ownership networks, the impact of trade policies with network perspectives, design of tariff and non-tariff measures under trade agreements, domestic industry relocation, evaluating the effects of government subsidies on firms’ long-term innovation, and design of subsidy strategies for supply chain stability and resilience. We believe research on supply chain resilience will thrive in the years ahead, better supporting firms’ and governments’ decisions.

## 1 Introduction

In recent years, global supply chains are extensively and deeply affected by events such as trade wars, the Covid-19 pandemic, and the Russia-Ukraine War. Facing frequent supply chain disruptions, firms have realized that traditional efficiency-oriented management is no longer sufficient for supply chains to survive and thrive at such a time of uncertainty [1]. How to build resilient supply chains has thus become a highly concerned topic.

Resilience originates from material science and refers to the ability of a material to resist deformation. The concept has been borrowed by the community of supply chain management for a long time and many scholars have contributed to its conceptual definition [2], [3]. A well-accepted one among them was proposed by Vugrin et al. [4]. They formulate supply chain resilience as an integration of three system capacities: absorptive capacity, adaptive capacity, and restorative capacity. Although those definitions promote our understanding, they are still far from providing a comprehensive resilience measure or guidelines for resilience enhancement in practice.

Due to the current complex and volatile business and political environments, firms as part of large industrial systems are not able to manage many supply chain risks alone. Modern supply chains have evolved to be large-scale complex network systems with heterogeneous nodes, tangled interconnections, uncertain structures, and dynamic interaction [5]. There is a need for planning, coordinating, and investing at broader system levels such as industrial sectors and countries to improve supply chain resilience [6], [7]. Correspondingly, supply chain researchers should go beyond simple supply chain structures by adopting a network perspective.

Most major powers have indeed proposed strategies to improve supply chain resilience and security. In 2012, the U.S. government released the *National Strategy for Global Supply Chain Security*, highlighting the concept of supply chain resilience and security for the first time. Subsequently, the *National Security Strategy Report*, the *Supply Chain Security Rules of the United States* and the *National Advanced Manufacturing Strategy* issued by the U.S. government have frequently mentioned the importance of enhancing supply chain resilience and released policies to reduce supply chain vulnerability. China has also repeatedly stressed the necessity to ensure industrial and supply chain security from a strategic standpoint and proposed to build a modern supply chain system with resilience. In 2022, China, together with other five countries, proposed the *Initiative of International Cooperation on Resilient and Stable Industrial and Supply Chains*. In addition, Japan, South Korea, and other countries have also issued bills and reports on supply chains, aiming to guide policies related to supply chain resilience and national security. In these reports, governments propose goals and general guidelines. However, how to achieve those goals by engaging in regional or global partnerships (i.e., macro perspectives) and by providing the right incentives to corporate entities or researchers (i.e., micro perspectives) is yet to be studied.

Thus, a network perspective and inclusion of more stakeholders such as governments are necessary to be studied in either evaluating supply chain resilience or proposing resilience improvement strategies. Section 2 reviews the related literature on identifying vulnerable nodes & links, risk propagation modeling, network resilience measures, and disruption mitigation strategies, and also discusses gaps and future research opportunities. Section 3 reviews the literature on governments’ roles in global supply chains (i.e., an external circulation perspective) and in incentivizing firms’ R&D and stimulating domestic demand (i.e., an internal circulation perspective), and proposes future research on policies evaluation and strategies to improve supply chain resilience.

## 2 Network perspective

As the famous proposition goes, *more is different* [8]. From this point of view, indiscriminate applications of firm-specific methodology to network settings risk committing the atomistic fallacy (i.e., overgeneralizing results at the individual level to the group level) [9]. To avoid such mistakes, we need to re-examine established progress in the literature and recognize research gaps.

In this section, we discuss supply chain network resilience in the logic that flows from local to global and from cognition to practice. Since most disruptions start from a local region and then ripple around the whole network, detecting network bottlenecks and characterizing risk propagation are of great importance (Section 2.1). On top of that, a global view of supply chain network resilience relies on its rigorous definition and systemic measures (Section 2.2). Moreover, supply chain network resilience is an application-driven field in essence. How to deal with disruptions proactively and reactively is a focal issue in practice. Hence disruption mitigation strategies are also combed through to target those that are promising to be extended to the network setting (Section 2.3). We have summarized the framework of this section in Table 1.

**Table 1:** **Framework of**Section 2.

Topics	Sections	Contents
Detecting bottlenecks	Section 2.1	Methodologies from SNA
		Bayesian networks
		TTR/TTS
Characterizing risk propagation	Section 2.1	Epidemiology models
		A default propagation model
Measuring resilience at a network level	Section 2.2	Network connectivity
		System performance variations over time
		An example of network resilience measure
Mitigating disruptions in supply chain networks	Section 2.3	Network topology design
		Redundancy
		Flexibility

### 2.1 Bottleneck detection and risk propagation

As a special case of networks, supply chain networks share some common properties with networks in other domains, and thus, researchers have borrowed methodologies in graph theory (GT) and social network analysis (SNA) [10], [11]. By defining nodes as firms and edges as material flows, measures of important nodes in GT/SNA (e.g., betweenness centrality, closeness centrality, hub and authority centrality, etc.) are naturally applied to bottleneck detection tasks [12], [13]. However, direct applications might ignore inherent heterogeneity in supply chain networks, possibly underestimating the potential risks from those seemingly inconspicuous but indispensable entities.

To capture information besides static supply relationship, researchers build Bayesian networks to quantify the impact of disruptions at network elements [14], [15]. Typically, elements are modeled either disrupted or fully operational. Conditional disruption probabilities that reflect the dependence among neighboring elements need to be extracted from historical data or expert knowledge as inputs to the model. Through Bayesian inference, we can calculate the change in the focal firm’s disruption probability when an upstream element is disrupted. The bottlenecks are identified as the entities exerting the most impact on the focal firm. Although Bayesian networks offer an inviting theoretical framework, they are hard to implement in practice since conditional probabilities of disruptions are typically unavailable.

Time-to-recovery (TTR) and time-to-survive (TTS) are more practical methods to find the most vulnerable suppliers [16], [17]. TTR means the time for a particular node (a supplier, a distribution center or a transportation hub) to be fully restored after a disruption. By removing one node a time from the supply chain network during the time span of TTR, we can calculate the unavoidable performance loss (measured in lost sales or revenue) caused by the disruption of that node. The entities with the largest risk exposure are identified as network bottlenecks. Typically, no historical experience is available and TTRs of suppliers (or other entities) can only be reported by themselves. Hence, it is likely for them to understate TTRs to get manufacturer’s preference. To cope with this bias, TTS is proposed as a supplement. TTS of a node refers to how long the supply chain network can still serve customer demand after the node is disrupted. The nodes with short TTSs are those critical entities whose TTRs need more attention and more accurate estimation. Although TTR and TTS are useful and practical in revealing the upstream bottlenecks of a focal firm, bottleneck detection in a (national or transnational) cross-industry supply chain network, a task more valuable but difficult for country-level policy makers, remains to be solved.

So far, we have reviewed some methods targeting network bottleneck detection. Ideally, we should detect bottlenecks in time and avoid potential disruptions in advance. Unfortunately, disruptions as low-probability events cannot be avoided completely, and once a disruption occurs, policy makers try to minimize its impact by affecting the risk propagation process. Another motivation for risk propagation characterization is to detect the most “infectious” nodes in the network. Once these nodes are affected or disrupted, the cascading failure may paralyze the supply chain network. Therefore, characterizing risk propagation processes is necessary.

To characterize risk propagation processes, researchers learn from epidemiology models (e.g., Susceptible-Infectious-Susceptible model) that describe infectious diseases propagation with differential equations. The characterization of health state and immunity measures vary in the literature. Some model the infectious rate and recovery rate to depend on the ability to collect information about customers and suppliers [18] and others argue that whether the susceptible supplier is domestic or foreign affects the infection rate [19]. These seemingly arbitrary selections of immunity measures and difficult estimation of parameters cast a shadow on their applications in practice. Moreover, how to capture unexpected black swans also poses a challenge.

Birge et al. [20] model risk propagation through undelivered orders and their impact on firms’ net worth. A firm’s net worth means its initial equity plus sales revenue minus the sum of production cost and safety stock cost. When a firm’s net worth is negative, it will default, which leads to a disruption. The default affects its buyers via unfulfilled demand and its suppliers through unreceived payments. To mitigate the adverse effects, the former will seek for alternative suppliers, and the latter will turn to secondary markets to reroute those undelivered orders. If their contingency actions are still not enough to absorb the disruption impact, contagious defaults will ensue. The authors have proved the existence of a unique supply chain network general equilibrium. Moreover, they propose an algorithm to trace the default propagating. Overall, their characterization is theoretically sophisticated and sets a milestone for disruption propagation modeling in supply chain networks. However, they assume fully rational firms and frictionless switching, which may not be met in reality.

For more modeling techniques, we refer interested readers to Bier et al. [13]. Detecting bottlenecks and characterizing risk propagation are essential for enhancing supply chain network resilience. They help to offer early warnings so that vulnerable entities can be fortified in advance. They are important in practice while challenging in theory. As we have seen, many methods have been proposed. However, the research community is far from reaching a consensus on how to tailor those models when taking a cross-industry, national, or even global view. To fill the gap, we propose some promising directions for future research.

First, it is necessary to derive a non-probabilistic framework from the network perspective. Since it is extremely hard to estimate disruption probability, practical methods to detect bottlenecks or characterize risk propagation should be non-probabilistic. TTR and TTS are such methods that have been successfully applied to firm-specific risk management [21]. However, it remains understudied in how to derive a non-probabilistic framework under the network setting. Second, it is important to develop methods targeting incomplete information. A firm in the network may have information access to its first-tier suppliers, but that to higher-tier suppliers is typically unavailable. Likewise, policy makers may have partial information of the industry networks, but are typically far from understanding the complete topology of the network and the interactions of firms. In such cases, lack of full information can mislead their policy making. Hence, more attention should be paid to methods of dealing with incomplete information. In particular, industrial network construction by aggregating information from multiple isolated parties is fundamental and critical for bottleneck detection and industry planning. Third, it is significant to design customized tools for different types of disruption. Spontaneous disruptions differ in nature from those caused by deliberate attacks. Moreover, disruptions that originate from various risk factors, such as supply shortage or disrupted cash flow, are also disparate. This suggests us to manage different risks with customized tools. Therefore, hybrid methods may be more promising in identifying network bottlenecks and characterizing risk propagation.

### 2.2 Definitions and measures of network resilience

Compared to locating susceptible entities in a supply chain network, defining and measuring resilience in a comprehensive way are even more tricky and understudied. It is necessary to clarify the scope of our review before any further discussion. First, the resilience that we stress in supply chain networks differs remarkably from that in general network science [22]. The latter emphasizes tipping points and phase shifts, which differ to some extent from our concerns. Second, we regard resilience as the capability to protect supply chain networks from disruption risks rather than operational risks. Thus, the studies focusing on traditional risk management are also beyond our discussion [23]. Third, we differentiate network-level studies from firm-specific ones in that they vary in risk nature and remedies [24].

The definition of resilience is typically built on concepts of disruptions. Kim et al. [9] distinguish disruptions at a network level from those at a node/arc level. They model supply chain networks as digraphs where materials flow from the source node(s) to the sink node. Disruptions at a network level are then described as a situation where there no longer exists a path between the source node(s) and the sink node. Hence, the resilience of a supply chain network is accordingly defined as the probability that random removal of elements does not result in a network level disruption. Although this measure steps towards a network view, its intrinsic static perspective should be carefully examined. For example, Kim et al. [9] conclude that network structures with power laws (20% of the nodes (hubs) connecting to the other 80%) possess higher resilience. However, when risk propagations are considered, hubs tend to be more susceptible and contagious to the periphery, possibly subject to higher frequencies of disruptions.

Besides network dynamics, nodes heterogeneity also raises the bar for measuring resilience. Zhao et al. [25] classify nodes into categories based on their functions. They point out that in military supply chain networks, some nodes like support units are indispensable to other regular battalions. Hence, the resilience metrics should differ from existing concepts in GT/SNA and be modified accordingly. For example, they replace traditional LCC (the largest connected component) with LFSN (the largest functional sub-network), which requires any pair of nodes connected as well as at least one supply node available. Their characterizations take heterogeneity into account in some sense, whereas node functions vary in not only types but also magnitudes. How to incorporate the heterogeneity quantitatively still obsesses researchers.

Another group of resilience measures is based on system performance variations over time. When a disruption happens, the system performance level will first drop and then climb. The former phase manifests the absorptive capacity of the system while the latter phase indicates its adaptive/restorative capacity. Some indices including rapidity (the average slope), performance loss (difference of integral), and recovery ability (performance ratio) are constructed accordingly [26]. There have been numerous pieces of research studying the integration or improvement of these indices [26], [27]. However, predicting performance evolvement before a disruption happens is remarkably challenging, especially for intricate supply chain networks. Thus, before this framework can apply, other modeling techniques must be incorporated. To be more specific, factors such as the network structure, inventory distribution, and capacity distribution should be considered.

Birge et al. [20] provide a measure that incorporates network structures and inventory distribution. In their model, firms can hold safety stock in advance to mitigate potential default impacts of their suppliers. When fundamental disruptions happen, they will switch demand or reroute supply as we have introduced in Section 2.1. Given exogenously specified inventory distribution, each firm will maximize its ex post net worth to determine the optimal switching/rerouting quantity. Then a unique general equilibrium for the supply chain network exists and can be derived by an algorithm. The authors argue that higher inventory level will reduce firms’ out-of-stock risk. Therefore, they aggregate inventory costs and switching demand costs of all firms, and use their percentage reduction as the network resilience measure. Note that their measure is a random variable due to the randomness in demand, production cost, and disruption occurrence. The stochastic setting may make their measure, although sophisticated in theory, relatively difficult to be applied in practice.

Overall, there are few existing works measuring resilience from a network perspective. Since locally optimal decisions are insufficient for managing risks at an aggregate level [28], researchers should pay more attention to resilience measures at a network level. We suggest the following directions for further exploration. First, it is critical to derive a non-probabilistic measure that incorporates dynamics. As we have mentioned above, typically a non-probabilistic resilience measure is more applicable in practice. Since dynamics are indispensable to resilience measures, incorporating dynamics into a non-probabilistic framework is critical. Second, it is beneficial to establish a unified framework for supply chain network resilience. A unified framework paves the way for resilience enhancement. It allows more consistent evaluation and comparison of mitigation strategies so as to better guide managers’ decisions. Although diverse measures exist in the literature, a unified framework might be possible if we take a broader perspective. For example, Behzadi et al. [29] propose a framework for restorative capacity. They categorize various measures into three classes: TTR (time to recover), RL (recovery level), and LPR (loss of performance while recovering). On the other hand, Birge et al. [20] focus on adaptive capacity. These measures can further be unified at a higher level.

### 2.3 Mitigation strategies for network disruptions

A large literature has studied both proactive and reactive strategies for supply chain disruption mitigation [30], [31]. We will present a sketch of their works as a starting point for further extension to network settings. We exclude business principles such as agility, velocity, corporate culture, etc. from our discussion [32], because we try to focus on execution level strategies. Nevertheless, we highly recognize their effectiveness as valuable principles, and advocate their continued applications, especially in combination with other measures, to network settings. Moreover, we do not include shift-style strategies such as insurance. They shift the financial loss away from specific firms, but the damage to the supply chain network lingers on. The proactive and reactive strategies that we consider promising in network settings are detailed respectively in the following paragraphs.

Among proactive strategies, supply chain network topology design (e.g., network site selection) is a structural-level strategy. Network resilience is highly contingent on its topological characteristics such as density, complexity, and the number of critical nodes [33]. For example, Blackhurst et al. [34] provide an empirical framework to prove that supply chain resilience is inversely related to network density. Hence strategies like supplier geographical segregation are put up accordingly [35]. The literature related to facility locations is reviewed in Snyder et al. [24]. Typically, the problem is formulated as supplier or facility selection aimed at minimizing the weighted sum of operational costs under normal and disrupted scenarios. However, few of them consider the correlation among suppliers’ risks which potentially leads to extensive simultaneous failures in the supply chain network. In addition, the majority of them focus on a single product and assume no higher-level suppliers, whereas multi-echelon structures are more reasonable from the network resilience perspective.

Redundancy may be the group of proactive methods most widely employed in the literature. It refers to adding buffers to supply chains to alleviate disruptions and includes broad practices such as inventory, multi-sourcing, alternative transportation modes, and so on. Most relevant studies focus on optimal inventory levels to balance efficiency and resilience. Typically, disruption scenarios and their respective probabilities are exogenous for minimizing overall ex-ante costs [36], [37]. However, the enumeration of unforeseen black swans and estimations of disruption probabilities both appear challenging. Furthermore, Simchi-Levi et al. [38] point out that the optimal inventory levels vary according to the network structures, so there are no universal rule-of-thumb methods for inventory allocation. To handle the correlational structure between different disruption events, Gao et al. [39] propose a conic program formulation for network inventory positioning. Nevertheless, the relaxed model that they solve deviates from the exact one, resulting in potential discrepancies between the solutions.

Flexibility is another frequently mentioned maneuver to achieve supply chain/network resilience, yet its connotations vary in different scenarios. A general understanding is that it measures the number and heterogeneity of alternative options. It differs from redundancy in that it can be applied reactively as well as proactively. Sourcing flexibility (e.g., multiple sourcing with backup suppliers) is a typical proactive flexibility. Process flexibility (e.g., utilizing a production facility to produce multiple products) is another frequently seen proactive flexibility [40], [41]. When demand of some products surges or certain suppliers are disrupted, sourcing or process flexibility allows firms to take recourse action by applying the built-in flexibility to absorb risks. The design of proactive sourcing flexibility or process flexibility can also be structural-level decisions. Hence, proactive flexibility design has some overlap with supply chain network topology design. For supply chain networks with multistage structures, Graves and Tomlin [42] propose a flexibility measure that lays the foundation for supply chain resilience improvement. The effectiveness of proactive flexibility rests on assumptions that the disruptive events are not systematic or the impact scales are not excessive. However, in practice, the demand for different products can be highly correlated and they may require shared capacities at the same time [43], in which case, the proactive process flexibility does not help much. Similarly, when there is an industry-wide shortage or a ban on the supply, proactive sourcing flexibility such as multiple sourcing or dynamic emergency response is ineffective [44]. In such situations, firms may resort to reactive flexibilities.

Reactive flexibility refers to firms’ capability to take recourse actions to construct ad hoc supply chains for immediate needs after disruptions. Such flexibilities enable firms to transfer capacities and resources to products faced with a surge in demand, or to obtain outside support to restore operations. Müller et al. [45] stress the central role of dynamic capabilities in achieving agility for building ad hoc supply chains. Besides that, for unforeseeable disruptions, firms with various backgrounds need to discover their own advantages in bridging the gap between target supply chains and their existing ones. For example, after the Covid-19 outbreak, BYD Auto redesigned its automobile manufacturing process to produce face masks. Within three months, their capacity increased to 50 million per day, which not only secured stable medical supplies in the market but also paved the way for other industries’ production reopening. The successful establishment of BYD’s reactive process flexibility roots in implicit linkages between the two seemingly irrelevant supply chains. It turns out that polypropylene, a critical raw material for face masks, is also utilized in auto manufacturing, and the aseptic conditions required for face mask processing can be easily satisfied in auto production workshops. This example implies, to build reactive flexibility, firms need dynamic capabilities and explore hidden connections within the supply chain network.

In our opinion, proactive strategies, including supply chain network topology design, redundancy, and proactive flexibility, help avoid, postpone disruptions, or alleviate the impacts of the disruptions. But they may fail when the magnitude of demand or supply shocks goes beyond certain thresholds. Reactive flexibilities, by contrast, are applied in a recourse manner based on realizations of new information and disruption outcomes and are promising to guide us out of the woods through a new trail. However, reactive flexibilities oftentimes are not readily there before disruptions. Their development requires firms’ creativity and connectability with other supply chain entities. Knowledge sharing among network entities is also critical. As such, a network view is essential for firms to identify potential reactive flexibilities. Huge gaps still exist between the existing supply chain research and future research with a network perspective. Proactive strategies have been studied extensively in the existing supply chain literature, but mostly not under network settings. The reactive strategies, in particular reactive flexibility, are understudied and call for further explorations in supply chain networks.

We also identify some more concrete directions for future research. First, we should distinguish strategies for systematic risks from that for idiosyncratic risks. As Osadchiy et al. [46] have shown, flexibility strategies targeting idiosyncratic noises are less valuable for systematic risks. Hence, differentiating two types of risk and tailoring corresponding strategies are necessary. Second, the optimality of a mitigation strategy relies on the resilience measure we adopt [29]. Among all possible resilience measures, we should figure out the most reasonable measure in certain situations and correspondingly design the most effective strategies under that measure. Third, methods that balance efficiency and resilience are worthy of further exploration. Risk mitigation strategies are typically accompanied with costs, which are against the efficiency goal. Several works have modeled the trade-off as a two-stage stochastic programming [36], [37]. However, probability estimation for all scenarios is extremely hard. Therefore, how to balance them in a more sophisticated way calls for more innovative ideas.

## 3 Incorporation of more stakeholders

In recent years, political factors such as the U.S.-China trade relationship and the Russia-Ukraine war play greater roles in shaping multinational firms’ global supply chain decisions and supply chain resilience. Traditional supply chain research that mostly focuses on only firms’ activities is not sufficient to inform firms’ operational resilience in such complex environments. This is because countries or governments play more active roles to influence certain industries’ competitiveness and resilience. Therefore, it is necessary to incorporate more stakeholders’ decisions and their joint impacts in supply chain research, in particular, resilience evaluation and improvement.

For instance, many countries provide incentives to encourage their enterprises in key industries such as semiconductors and critical medicines to move back or move to allied countries. This directly affects China, one of the most popular overseas sourcing destinations for global organizations. To adapt to the new environment, China has formulated the equally important internal and external circulation strategies. In terms of external circulation, i.e., China’s economic links with the outside world, the government actively engages in trade agreements. Regarding internal circulation, it is critical to provide the right incentives to motivate firms’ research and development (R&D) investments, stimulate domestic demand, and optimize industry distribution across the nation. Such extensive interventions from the governments of different countries deeply influence the firms’ global supply chain design, investments, and operations.

**Fig. 1:**
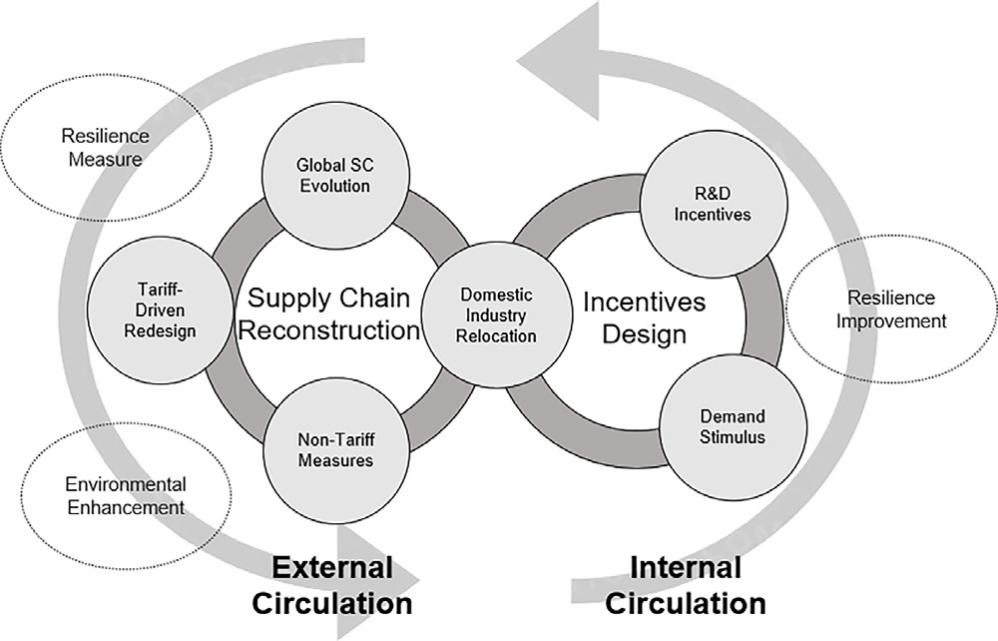
**Framework of**Section 3.

In the following, we first discuss topics on global supply chains, including supply chain evolution and the underlying driving forces, the impact of tariffs and non-tariff measures on global supply chain structures. Then we review the existing research on governments’ incentive design and discuss research opportunities on government policies that enhance firms’ R&D activities and stimulate domestic market demand. Incorporating more stakeholders such as governments and industrial sectors will be a major distinctive feature of such studies compared to the most existing supply chain research. The framework of this section is shown in Fig. 1. We discuss how to measure global supply chain resilience and improve the resilience from the perspectives of supply chain reconstruction and incentives design.

### 3.1 Supply chain reconstruction

One key feature of re-globalization is regionalization. To strengthen their influence and supply chain resilience, both developed countries and emerging economies have incentives to establish regional trade agreements. Currently, there are three representative regional trade agreements: RCEP (Asia and Oceania), The United States - Mexico - Canada Agreement (USMCA) (previously North American Free Trade Agreement(NAFTA)), and the Treaty of Rome (Europe). Across these regions, there are trade agreements such as Free Trade Area of the Asia-Pacific (FTAAP) and Comprehensive and Progressive Agreement for Trans-Pacific Partnership (CPTPP), and the recently proposed Indo-Pacific Economic Framework (IPEF). China has signed 19 free trade agreements with 26 countries and regions, covering Asia, Oceania, Latin America, Europe, and Africa. As the global trade landscape changes fast, many new challenging problems emerge, which creates unprecedented research opportunities. We first explore resilience evaluation from the macro perspective and then review the interactive studies from the macro policy perspective (including international policies such as tariff and non-tariff measures, and domestic industrial transfer policy) and the micro firm perspective.

#### 3.1.1 Global supply chain evolution

Constructing global supply chain networks using trade and world input-output data serves as a foundation to analyze the evolution and the underlying driving forces of trade relationships within regions and across regions. Is there an increase of within-region trade relative to cross-region as expected, and by how much? What industries change most significantly? For example, Timmer et al. [47] measure cross-border supply chain fragmentation and show the fragmentation of supply chains adds 0.44 points to the global trade elasticity before the 2008 financial crisis, while only 0.05 points after that. Charoenwong et al. [48] empirically investigate how firms restructure global supply chains under the trade and economic policy uncertainty. They find as the uncertainty of the U.S. trade policy increases, firms with high domestic revenue shares reduce the number of foreign suppliers, whereas firms with a majority of foreign sales increase the number of foreign suppliers. Cheng et al. [49] explore the mechanism of the China-U.S. trade imbalance based on the SVAR model and find that the difference of comparative advantages in service trade is an important reason for the trade imbalance between China and the U.S. Hsu et al. [50] find regional value content requirements under the United States-Mexico-Canada Agreement (USMCA) are effective tools to encourage re-shoring and near-shoring by domestic firms.

By focusing on key industrial sectors and supply chains within a broader network, we are able to analyze the interdependence of supplies and demand across countries and regions, which may reveal to some extent the potential risks taking into account major-power relations. However, focusing on past trade data is far from enough to evaluate the resilience of supply chains. We also need to look into technology networks and companies’ ownership networks to uncover an industry’s weak link or node that is of low resilience. For example, in the semiconductor industry, when the U.S. launched the chip war against China, firms from multiple countries have been banned from selling semiconductor equipments to China. This is driven by the U.S. having a strong control power in the technology chain and the financial chain. In particular, the U.S. controls the core technology in key links of the industrial chain and has a large share of major semiconductor companies. Therefore, analyses from technology and ownership perspectives should have strong risk predictive power given a certain political or economic shock. Hence, studying global technology networks (e.g., the patent citation network) and firm ownership networks, and integrating these networks with trading networks would enable us to devise a more comprehensive resilience measurement framework. It is worth noting that there are some challenges in integrating the three-layers networks because of incomplete data. We may obtain more data by cooperating with relevant firms, and start with some important industries for research on supply chain resilience.

#### 3.1.2 Tariff-driven global supply chain redesign

In addition to risk and resilience evaluation from the macro perspective, there is a need for interactive studies from the macro policy perspective and the micro firm perspective. Under the all kinds of existing trade agreements, how should multinational firms redesign their global supply chains (e.g., global capacity deployment or sourcing destinations) by taking into account special rules and regulations along the tariff and non-tariff dimensions? On top of that, how should governments optimize tariff and non-tariff measures in trade agreements?

The effects of tariffs on multinational firms’ supply chain network design have received some attention from the academic community on operations. There is a stream of studies related to the effect of tariffs on supply chain networks of multinational firms. For example, Li et al. [51] investigate material sourcing strategies by taking into account tariff concessions through local content. Chen and Hu [52] investigate how tariffs affect the production layout of manufacturers. Cui and Lu [53] explore the impact of local component requirements on global sourcing and how to optimize local content requirement policy to maximize social welfare. Dong and Kouvelis [54] explore the impact of tariffs on firms’ global facility network.

Albeit the existing seminal works, the impact of tariffs on global supply chain structure is understudied. For example, the existing papers mostly focus on sourcing strategies, capacity planning, or selling strategies under frameworks involving two countries. However, due to the network structure of global supply chains, multiple countries are often relevant in global decisions. For instance, as the soybean tariff from the U.S. to China is raised, soybean exports from the U.S. to Brazil are increased and the soybean production capacity in Brazil is expanded [55]. Hence, at least three countries are active players in the soybean supply chain. How should soybean producers in the three countries react in terms of their production and selling strategies? Further investigation of the soybean processing supply chain would require a network modeling for not only soybean but also its downstream outputs such as soybean oil, soybean meal, and even the livestock that the soybean meal is fed to. Modeling the relevant important stakeholders, countries, and markets could enrich our understanding of the studied issues, but meanwhile would lead to a network model that may require new tools from multiple disciplines to analyze.

The existing tariff related supply chain studies usually consider deterministic tariffs. However, given the recent dynamic international environment, tariffs are highly uncertain. Wu et al. [56] investigate whether multinational firms should develop new contract manufacturers in a new region to overcome the uncertainty of future tariffs and find increasing uncertainty may lead the diversified development strategy to be more likely the equilibrium. Kouvelis et al. [57] show a higher raw-material tariff will reduce reshoring capacity investment, but an increase in finished-goods tariff can either increase or decrease reshoring capacity. These papers mainly take the perspective of multinational firms in global network design. In fact, many contract manufacturers in China also need to decide whether to build production facilities in either Southeast Asia or Mexico. An integrated view of multinational firms’ and contract manufacturers’ interactive global layout decisions deserves more future research.

#### 3.1.3 Non-tariff measures

Although tariffs have been reduced significantly in trade agreements, the growing use of non-tariff measures (e.g., quotas, requirements on sanitary and phytosanitary certificates, and technical regulations) for trade protection has led to a less transparent trade policy environment [58], [59]. Hence, understanding the impacts of non-tariff measures and negotiating non-tariff barriers besides tariffs in regional trade agreements become increasingly important.

There is a stream of literature related to the effect of non-tariff measures on trade flows. Saini [60] finds that over 60% of the value of India’s textiles and clothing exports has been affected by non-tariff barriers of major export partners. Chen et al. [61] show that unofficial non-tariff trade barriers account for 50% of the reduction in Chinese imports from the U.S. during the U.S.-China trade war between 2018 and 2019. However, it is worth noting that non-tariff measures do not always reduce trade. Timini and Conesa [62] point out that technical non-tariff measures have a positive impact on trade flows, potentially driven by improvements in consumers’ confidence and technical capabilities of Chinese exporters. Santeramo and Lamonaca [63] show that imposing maximum residue levels and ad valorem equivalent tends to increase trade volumes and technical barriers do not always limit trade.

Moreover, some studies investigate the impact of non-tariff measures on global value chains. Cadestin et al. [64] show that non-tariff measures will increase certain costs on global value chains and find non-tariff measures used by Latin American countries impose an additional cost on intermediate goods equivalent to a tariff of about 15%. Webb et al. [65] investigate the impact of non-tariff shocks on GDP and welfare. They find that approximately half of the non-tariff shocks are allocated to exporters and a half to importers and the partial liberalization by ASEAN countries of non-tariff measures can increase the GDP and welfare of all ASEAN countries. Korwatanasakul and Baek [66] show non-tariff measures have negative effects on global value chain participation and the impact of non-tariff measures is larger than that of tariff measures.

Although non-tariff measures have drawn research attention in terms of their impacts on trade volume and economic performance, it is understudied from the perspective of global supply chain design. Is there any empirical evidence that non-tariff measures drive certain industries’ location choice and technology investment? For industries that are heavily affected by both tariffs and non-tariff measures, it is worth studying how firms should react to their joint impacts for improving their ability to absorb and adapt to shocks. From a macro perspective, how do non-tariff measures affect trade flows? How to identify key links and countries that affect smooth trade flows? How to design and negotiate non-tariff barriers in regional trade agreements with other countries to enhance the resilience and security of supply chains? Addressing such questions with a network scope is expected to result in fruitful research outcomes.

#### 3.1.4 Domestic industry relocation

The literature on China’s domestic industry relocation mainly focuses on feature characterization and effectiveness evaluation. Zhou and Zhou [67] investigate whether the relocation demonstrates an industrial clustering effect and find that labor-intensive manufacturing industries are easier to form cyclic accumulative effects. Ruan and Zhang [68] empirically test whether there is the transfer of textile and garment industries from the eastern region of China to the central and western regions. Chen et al. [69] apply the spatial Durbin model to show that industrial relocation has a markedly positive effect on improving industrial land use efficiency. Li et al. [70] find that the establishment of the National Industrial Relocation Demonstration Zones reduces the energy efficiency of the demonstration zones and cannot promote industrial structure advancement. To the best of our knowledge, it is yet to be studied which industries should move to the midwest areas and how to incentivize the relocation for different industries.

Taking a network perspective, trying to answer the following questions serves as a good starting point to further the domestic industry relocation studies: How to model a network based on regional and industry properties? How to use the existing performance of industrial relocation practices to guide the optimization of the layout of different industries in different regions? How to optimize the path of industrial transfer? How to encourage nodes (regions) on the transfer path to cooperate so as to leverage their comparative advantages and achieve resource allocation efficiency? Nationwide network modeling faces the following challenges: First, data availability. We would require information such as regional human resources, labor, technological capability, natural resources, relative industry competitiveness, infrastructure, etc. Depending on data availability, we may model cities, provinces, or other definitions of regions as nodes. Second, the interactive behaviors. Since industrial transfer involves complicated interest reallocation, it is inevitable to model gaming among participators. How to model nodes behaviors and games in a network is a common challenge most network-based supply chain research needs to tackle and we believe it will advance the network theory as well.

### 3.2 Incentives design

Global supply chains have suffered from great uncertainties in recent years. A stream of literature investigates the measures of firms for improving supply chain resilience, such as multiple sourcing [71], [72], [73], inventory for risk mitigation [74], [75], insurance [76], [77], and so on.

However, it is difficult for firms alone to deal with extreme shocks in industrial and supply chains. Supports from governments are particularly important in these scenarios. Accordingly, we need to address questions such as how to design relevant policies and mechanisms that enable the government and enterprises to work together to enhance supply chain resilience. We focus on the design of incentives provided by the governments to enhance firms’ R&D capability and to stimulate domestic market demand for ensuring supply chain security.

#### 3.2.1 R&D incentives

An unstable supply of key components or shortages of critical equipment due to international relations may lead to disruptions of supply chains and may even endanger firms’ survival. In particular, the technology blockade imposed by the U.S. government has caused serious concerns over China’s industrial and supply chain security, which necessitates greater investments in core technologies in China. However, R&D investments are capital intensive and face high uncertainties in the outcome. How to motivate firms to improve their innovation capabilities becomes an important topic for the Chinese government.

There is a stream of literature on the effects of government subsidies on R&D outputs, but the results are mixed. Some studies point out that government subsidies can directly make up for the capital shortage of firms, and boost R&D outputs of enterprises [78]. Furthermore, government subsidies can serve as a positive signal of firms’ capabilities, which can help firms attract larger-scale investment [79], [80]. However, other studies find that government subsidies may lead to rent-seeking behaviors [81] and crowd out firm-financed R&D spending [82]. In addition, Liu et al. [83] identify an inverse U-shaped relationship between subsidies and firms’ innovation. Although the relationship between government subsidies and firms’ innovation has received much research attention, most studies focus on short-term impacts on firms. However, since the effects of subsidies focusing on core technologies are hard to manifest in the short term, with the objective of achieving breakthrough improvements in core technologies and hence enhancing supply chain resilience, we have to extend our studies’ time horizon and evaluate the effects of government subsidies on firms’ long-term innovation.

In terms of the choice of subsidy strategies to encourage R&D, Zuo et al. [84] investigate three options for R&D subsidies including immediate subsidy, delayed contingent decision, and no subsidy. They find that compared with the mechanism of two options (i.e., immediate subsidy and no subsidy), the three-option mechanism can help governments identify firms with high growing potential and achieve reasonable resource allocation. Chen et al. [85] explore the impact of different subsidies (per-unit production subsidy and innovation effort subsidy) on sustainability innovation and point out that the government should never use both types of subsidies simultaneously for any cost-reduction R&D effort. Xie et al. [86] investigate the effectiveness of production subsidy and R&D innovation effort subsidy on the vaccine product and find the per-unit production subsidy is better in terms of social benefits for high potential demand or a low level of risk aversion of consumers. In the above papers, the main objectives considered by the government are social welfare and consumer surplus, and the goal of most enterprises is to maximize their profits. However, the objectives of the government and firms will change when facing supply disruption risks and industrial and supply chain security problems. That is, the government may be more concerned about supply chain stability and the firms pay more attention to their capability of dealing with unexpected shocks. Thus, it is necessary to consider how to design subsidy strategies under the new objectives. Furthermore, the difficulty of innovation varies across industries, and how to design appropriate subsidies contingent on industry features deserves more future research.

In addition to directly subsidizing manufacturers, the government may also subsidize its supply chain partners. For example, there are both downstream consumption subsidies and upstream direct R&D subsidies in the new energy vehicle industry. The purpose of the consumption subsidy is also to promote industry development. In addition to downstream consumers, there are many upstream suppliers. However, upstream suppliers lack core technologies and products are more likely to be homogeneous, leading to vicious price competition among them and low motives for innovation. It remains to be studied how the government can screen suppliers with potential innovation capability for subsidy, so as to promote the collaborative innovation of the whole industrial chain. Due to the possible spillover effects of innovation across different stages of a supply chain, it is also meaningful to investigate the transmission mechanism of subsidy effects and choose the right party to subsidize.

In environments with technology blockade, it is particularly important for firms to use extensive international resources and develop international cooperation to enhance their technical capabilities [87], [88]. However, during the cooperation, there is a caution that firms may lose control over the joint innovative achievements. To provide a good institutional environment for innovation, governments should pay more attention to the policies related to cross-border technology transfer and intellectual property protection. On one hand, regulations are necessary to protect local firms’ rights over the joint ownership of the innovation outcome. On the other hand, the restrictions over cross-border technology transfer should not be overly strict to discourage foreign firms’ participation in cooperative innovation. More research is needed on the design of technology protection policies and subsidy policies to encourage local firms to participate in international cooperation. Another caution in international cooperation is that firms rely on foreign advanced technology and lose the ability to develop core technologies independently. How to incentivize firms’ independent technology development remains a topic for future research.

#### 3.2.2 Demand stimulus

In economic downturns, stimulating consumption and expanding domestic demand are important drivers for economic growth. In the wake of the Covid-19 pandemic, services such as catering, retail, tourism, and entertainment have been severely affected, and many small and medium-sized enterprises are on the verge of closure. Governments in different countries have adopted policies to stimulate consumption, such as tax rebates, cash payments, and consumption coupons.

There is a stream of studies investigating the impact of stimulus policies on consumer behavior. Previous studies find the marginal propensity to consume is between 0.1 and 0.9 for tax rebate or government cash payment [89], [90], [91], [92].^2^ In response to the Covid-19 pandemic, governments have also issued stimulus policies for consumption. Baker et al. [93] find the marginal propensity to consume is 0.25 to 0.4 for the U.S. CARES cash payments. Under the stimulus policy, the increase in durables spending is small, while the increase in food spending is large. Kaneda et al. [94] explore the effect of Japan’s cash transfer program on household consumption and find the consumption of food, necessities, and services rises significantly, but that of durable goods does not. Feldman and Heffetz [95] investigate a one-time and universal Covid-19 cash transfer program in Israel and find financially weaker respondents use the cash for debt payments.

Compared to tax rebate and cash payments, there are fewer studies investigating the effect of coupon/voucher program on stimulating consumption, since coupon program is less applied by the government. In 1999, the Japanese government distributed shopping vouchers worth 20,000 yen to families with children under 15 and more than half of the elderly. Hsieh et al. [96] find coupons actively promote the consumption of semi-durables, but do not affect spending on nondurables or services, and the marginal propensity to consume semi-durables is between 0.1 and 0.2. Regarding 2009 Taiwan shopping vouchers worth NT $3,600 of two denominations (including six NT $500 coupons and three NT $200 coupons to each individual), Kan et al. [97] find the marginal propensity to consume is about 0.25 and discounts offered by vendors to voucher users improve the effectiveness of the program. To mitigate the economic shocks of the Covid-19 pandemic, the Chinese government mainly issued digital coupon programs to stimulate consumption. Xing et al. [7] investigate the effect of the Shaoxing shopping coupon program targeting particular industries and find coupons lead to an immediate increase in weekly consumption, with an additional 3 yuan in out-of-pocket spending for every 1 yuan of government subsidy. Similarly, by studying the effect of Hangzhou’s digital coupon program without industry restrictions on consumer spending, Liu et al. [98] find that 1 yuan subsidy can drive 3.4–5.8 yuan of excess spending. The stimulus effectiveness of coupons by the Chinese government is much higher than tax rebates, cash payments, and vouchers in other countries, probably because of the following designs of coupons. First, the use of coupons is limited to a minimum amount of spending, such as spending 40 and getting 10 off. Second, the expiration dates of coupons motivate loss-averse consumers to speed up their spending. Third, the randomness of obtaining the coupons makes the consumers receiving the windfall more willing to use coupons.

The above reasoning for the effectiveness of Chinese coupons has not been tested. Rigorous empirical studies about the relationship between coupon design and effectiveness deserve more future research. Although the design of coupons is crucial, it has rarely been studied. The following questions are worth exploring: When should the government issue vouchers, to whom (all consumers, a specific group, or a random population), and in what amount? Which industries should have higher priority for coupon programs to be applied? Whether to limit the minimum amount of spending when using coupons? Whether should the government pay for vouchers or encourage firms to participate in co-payments? Careful examination of these questions serves as a foundation for successful coupon programs.

## 4 Conclusion

The U.S.-China trade frictions, the Covid-19 pandemic, and the Russia-Ukraine War posed great challenges for global supply chains yet created abundant research opportunities for supply chain researchers. Since the disruptions caused by such shocks are more systematic and the duration is harder to predict compared to natural disasters or other operational risks, we postulate two directions for future supply chain research: a network perspective and incorporation of more stakeholders such as governments that can influence industrial and supply chain performances at a high level.

From the network perspective, it is essential to stop propagation during disruptions and restore disrupted elements after crises. For the former goal, we need to target the network bottlenecks and figure out the spillover effects of disruptions. For the latter objective, we should find ways to measure and reinforce network resilience. Hence, we first sort out representative models for bottleneck detection and risk propagation. Based on them, we proposed three promising directions for future research: deriving a non-probabilistic framework from the network perspective, developing methods targeting incomplete information, and managing different types of disruption risk with customized tools. Second, we review definitions and measures of network resilience in the supply chain literature. We find existing studies are either probabilistic or unable to incorporate dynamics. We argue that a practical resilience measure should be both non-probabilistic and capable of capturing dynamics. We suggest constructing a unified framework for resilience measure that helps further resilience enhancement. Finally, we summarize several categories of disruption mitigation strategies and discuss possible extensions to network settings. Three promising directions are also identified: distinguishing strategies for systematic risk from that for idiosyncratic risk, figuring out the most reasonable resilience measure and the corresponding effective strategies under that measure, and balancing efficiency and resilience. We have listed the main future research suggestions from network perspectives in Table 2.

**Table 2:** **Main future research suggestions from network perspectives**.

	Main Future Research Suggestions
Bottleneck Detection and Risk Propagation	Derive a non-probabilistic framework from the network perspective
	Develop methods targeting incomplete information
	Manage different types of disruption risk with customized tools
Resilience Measures	Derive a non-probabilistic measure that incorporates dynamics
	Establish a unified framework of resilience measures
Mitigation Strategies	Distinguish strategies for systematic risk from those for idiosyncratic risk
	Figure out the most reasonable resilience measure and the corresponding effective strategies under that measure
	Balance efficiency and resilience

In terms of incorporating more stakeholders’ roles in the supply chain network decisions, we focus our discussions on the government’s role in global supply chains (i.e., an external circulation perspective) and in incentivizing firms’ R&D and stimulating domestic demand (i.e., an internal circulation perspective). We first review the existing research on global supply chains including supply chain structure evolution, tariffs, and non-tariff measures. Based on that, we suggest research topics including global supply chain resilience evaluation using trade networks, technology networks, and firm ownership networks, the impact of trade policies with network perspectives, design of tariff and non-tariff measures under trade agreements, and domestic industry relocation. Then we review the existing studies on the effects of government subsidies on R&D outputs and design of subsidy format and suggest future research topics including evaluating the effects of government subsidies on firms’ long-term innovation, design of subsidy strategies with the objective of supply chain stability and resilience, the transmission mechanism of subsidy effects across the supply chain, choice of the party to subsidize, and policy design in cross-border technology transfer and intellectual property protection. We summarize the main future research suggestions in Table 3.

**Table 3:** **Main future research suggestions taking into account more stakeholders**.

	Main Future Research Suggestions
Global Supply Chain Evolution	Global supply chain network construction and analysis using trade and world input-output data
	Evaluation and prediction of global supply chain resilience by integrating trade networks, technology networks, and firm ownership networks
Tariff-Driven Global Supply Chain Redesign	Interactive studies from the macro policy perspective and the micro firm perspective
	Global supply chain redesign by taking into account tariff uncertainty
	An integrated view of multinational firms’ and contract manufacturers’ interactive global layout decisions
Non-Tariff Measures	Understanding the impacts of non-tariff measures on global supply chain design
	Combinatorial effects of tariff and non-tariff measures on firms’ decisions
	Design and negotiate non-tariff barriers in regional trade agreements
Domestic Industry Relocation	Construction of a network based on regional and industry properties
	Optimization of the layout of different industries and the path of industrial transfer
	Incentives of nodes on the transfer path to cooperate
R&D Incentives	Design of subsidy strategies with the new objective of supply chain stability and resilience
	Incentives design for promoting collaborative innovations of industrial chains
Demand Stimulus	Empirical studies on the effectiveness of coupon design
	Design of vouchers (to whom, in what amount, in which industry, paid by the government or firms, etc.)

The postulated future research requires cooperation among researchers in multiple disciplines such as supply chain management, network science, economics, data analytics, and so on. As governments highlight the importance of industrial and supply chain resilience and security, we notice that scholars from areas other than supply chain management start to consider related issues on supply chain resilience. With more ideas and methodologies coming from different disciplines, we believe research on supply chain resilience will thrive in the years ahead, better supporting firms’ and governments’ decisions.

## Declaration of competing interest

The authors declare that they have no conflicts of interest in this work.

## Footnotes

2 The marginal propensity to consume is x if for each $1 received, consumers on average spend $x.
